# Prevalence of chronic kidney disease in Kazakhstan: evidence from a national cross-sectional study

**DOI:** 10.1038/s41598-023-42031-2

**Published:** 2023-09-07

**Authors:** Liza Nursultanova, Kairat Kabulbayev, Dinara Ospanova, Aigul Tazhiyeva, Ubaidilla Datkhayev, Timur Saliev, Shynar Tanabayeva, Ildar Fakhradiyev

**Affiliations:** 1 Kazakhstan’s Medical University “KSPH”, Almaty, Republic of Kazakhstan; 2 S.D. Asfendiyarov Kazakh National Medical University, 94, Tole-Bi Str., Almaty, 050020 Republic of Kazakhstan; 3 Al Farabi Kazakh National University, Almaty, Republic of Kazakhstan

**Keywords:** Diseases, Health care, Medical research, Risk factors

## Abstract

To date, there have been no large-scale national studies of the prevalence of chronic kidney disease in Kazakhstan. It includes the research based on the analysis of the estimated glomerular filtration rate (eGFR). The aim of this study was to investigate the population prevalence of CKD and associated risk factors in Kazakhstan. The cross-sectional study consisted of a nationally representative sample of n = 6 720 adults aged 18 to 69 from 14 regions and 3 major cities in Kazakhstan. The study covered the period from October 2021 to May 2022. The WHO STEPS questionnaire was used for the survey. For the diagnosis of CKD, creatinine levels in collected blood samples were measured to assess eGFR. Demographic characteristics were collected and studied. The total and adjusted prevalence of factors associated with the presence of CKD were calculated and analysed using logistic regression. 73.5% (n = 4940) of participants had normal eGFR, while 25.2% (n = 1695) had mild CKD (eGFR = 60–89 mL/min/1.7 m^2^). The overall prevalence of CKD with eGFR < 60 ml/min/1.7 m^2^ was 1.3% (n = 85), of which 0.2% (n = 15) had eGFR < 45 ml/min/1.7 m^2^. A mild degree of CKD was most often determined in residents of the East Kazakhstan region in 10.4%, and in 7.8–8.0% of cases. The majority of CKD patients was detected in the East Kazakhstan region and Almaty city, 15.3% and 10.6% of cases respectively. In mild and CKD with GFR < 60 ml/min/1.7 m^2^, the age of participants was 50–69 years in 61.5% and 78.8% of cases, respectively (*p* < 0.001). In addition to the association with the place of residence, a statistically significant relationship was found between the risk of developing CKD and underweight (OR 1.43, 95% CI (1.09–1.88), *p* < 0.001), as well as the presence of obesity (OR 1.24, 95% CI (0.99–1.53), *p* = 0.04). We observed the prevalence of CKD with eGFR < 60 ml/min/1.7 m^2^ at the level of 1.3%. However, a fairly large part of study participants had a mild CKD (25.2%). The results of this study can be used for the optimization of the doctors workload and the timely provision of care to patients with CKD.

## Introduction

Chronic kidney disease (CKD) is a major public health problem in many countries^1^. In 2019, there were over 697 million patients with CKD worldwide^2^. Patients with CKD have a higher risk of developing end-stage renal disease, which requires costly treatments such as dialysis and kidney transplantation, and this financial burden causes long-term physical and psychological complications^3^. Evidence from studies conducted across Canada, the US, Europe, and Australia indicate that CKD-related costs and outcomes varied significantly at different stages. From a health system perspective, moving from stage 3 CKD to stages 4–5 was associated with a 1.3- to 4.2-fold increase in costs, with the largest cost associated with terminal renal failure ($20,110–$100,593)^4^. It is predicted that by 2030 more than 70% of patients with end-stage renal disease will be residents of developing countries^5^. Moreover, CKD, even in its early stages, significantly increases the risk of developing cardiovascular disease^6^. According to some data, older, females with higher BMI, proteinuria and hyperuricemia, complicated by hypertension and diabetes, tend to be more susceptible to CKD progression^7^. Given its growing global burden, there is an urgent need to identify risk factors^8^ in order to develop targeted protection and control policies relevant to public health. In low- and middle-income countries, primary, secondary and tertiary prevention measures need to be implemented in collaboration with governmental and non-governmental organizations to stem this tide and help prevent negative outcomes from other non-communicable diseases that have similar risk factors to CKD^9^.

CKD prevalence is known to vary across countries due to differences in age, ethnic groups, survey policies, and eGFR calculation^10^.

Information on the prevalence of CKD using nationally representative data can serve as a guideline for determining the current prevalence of CKD. It can help to plan health care, allocate resources, and guide public health policies for the prevention, early detection and treatment of CKD^11^. The CKD risk factors, cardiovascular mortality in the general population, and public health policies may impact the prevalence of CKD^12^.

Nurtazina et al. conducted a study to assess the relationship between early renal dysfunction and lipid profile parameters in patients with hypertension on the territory of Kazakhstan^13^. However, this study was narrowed to the limited area (conducted only in one region), with the inclusion of only representatives of the Kazakh ethnic group. Another study examined the prevalence, morbidity, and mortality of patients on dialysis in Kazakhstan, however, using registry statistics, without prospective participation, and limited to a sample of patients on dialysis^14^. Thus, it should be noted that in Kazakhstan, no large-scale epidemiological studies of the prevalence of CRF among the population have been conducted yet.

In this regard, the aim of this study was to determine the population prevalence of CKD and related factors in Kazakhstan.

## Results

Among all respondents (n = 6720), n = 4401 were residents of the city, while n = 2319 were residents of the countryside. By gender, the number of men and women was almost the same, amounting to n = 3365 (50.1%) and n = 3355 (49.9%), respectively. The mean age of men was 40.1 ± 13.6 years, and that of women was 41.5 ± 14.1 years.

The general clinical and demographic characteristics of the study participants are presented in Table 1. By gender, among urban residents, men accounted for n = 2391 (54.3%), and women n = 2010 (45.7%). While, among the villagers, women accounted for n = 1345 (58%), and men n = 974 (42.0%). The average age of rural residents is 43.7 ± 14.0 years, while this indicator for urban residents was less and equal to 39.2 ± 13.5 years.

**Table 1:** General clinical and demographic characteristics of study participants.

Indicator	Urban n -4401		Rural n-2319	
	Male	Female	Male	Female
Gender (n, %)	2391 (54.3)	2010 (45/7)	974 (42)	1345 (58)
Age	39.02 ± 13,2	39.41 ± 13.8	42.6 ± 14.2	44.5 ± 13.9
	39.2 ± 13.5		43.7 ± 14.0	
BMI	24.4 ± 5.6	28.4 ± 6.4	25.1 ± 5.9	28.5 ± 6.5
	26.3 ± 6.3		27.15 ± 6.5	
Underweight	404 (9.2)		175 (7.5)	
Normal weight	1486 (33.8)		674 (29.1)	
Overweight	1298 (29.5)		696 (30)	
Obesity I degree	751 (17.1)		465 (20.1)	
Obesity II degree	462 (10.5)		309 (13.3)	
Education				
No school education	46 (1)		26 (1.1)	
Completed elementary (4 grd)	9 (0.2)		2 (0.1)	
Finished secondary (9 grd)	245 (5.6)		171 (7.4)	
Finished secondary (11 grd)	1115 (25.3)		676 (29.2)	
Higher	2111 (48)		961 (41.4)	
Master/Postgraduate/Doctorate	797 (18.1)		447 (19.3)	
7	64 (1.5)		12 (0.5)	
Refuses to answer	14 (0.3)		24 (1)	
Nationality				
Kazakh	2886 (65.6)		1488 (64.2)	
Russian	1028 (23.4)		522 (22.5)	
Uzbeks	95 (2.2)		107 (4.6)	
Ukrainians	79 (1.8)		31 (1.3)	
Uighurs	36 (0.8)		1 (0.01)	
Tatars	67 (1.5)		50 (2.2)	
Other	205 (4.7)		118 (5.1)	
Refuses to answer	5 (0.1)		2 (0.1)	
Marital status				
Single, not married	1148 (26.1)		379 (16.3)	
Married	2781 (63.2)		1666 (71.8)	
Married/married but living separately	34 (0.8)		20 (0.9)	
Divorced	252 (5.7)		134 (5.8)	
Widower/widow	125 (2.8)		100 (4.3)	
Is in a civil marriage	52 (1.2)		20 (0.9)	
Refuses to answer	9 (0.8)		0	
Smoking				
Yes	941 (21.4)		343 (14.8)	
	745 (16.9)	196 (4.4)	273 (11.7)	70 (3.0)
No	3460 (78.6)		1976 (85.2)	
	1646 (37.4)	1814 (41.2)	701 (30.2)	1275 (54.9)
In the past 12 months, how often have you had at least 1 standard drink of alcohol?				
Daily	13 (0.3)		7 (0.3)	
5–6 days a week	9 (0.2)		4 (0.2)	
3–4 days a week	36 (0.8)		11 (0.5)	
1–2 days a week	284 (6.5)		74 (3.2)	
1–3 days per month	521 (11.8)		151 (6.5)	
Less than once a month/Holidays	1243 (28.2)		509 (21.9)	
77	2295 (52.1)		1563 (67.4)	
eGFR type				
Stage 1	3294 (74.8)		1647 (71.0)	
Stage 2	1060 (24.1)		636 (27.4)	
Stage 3A	40 (0.9)		30 (1.3)	
Stage 3B	4 (0.1)		4 (0.2)	
Stage 4	1 (0)		1 (0)	
Stage 5	2 (0)		1 (0)	

According to BMI, normal weight was determined in urban and rural areas in n = 1486 (33.8%) and n = 674 (29.1%), respectively. While overweight was characteristic in almost the same 29.5–30% of cases for residents of the city and the village.

According to the level of education, in most cases, among the residents of the village, in n = 676 (29.2%) cases, respondents with completed secondary education (grade 11) prevailed in comparison with n = 1115 (25.3%) residents of the city. While n = 2111(48.0%). respondents from the city had a higher education, which was higher than the given indicators among the residents of the village, equal to n = 961 (41.4%). Whereas, respondents who received postgraduate education accounted for n = 797 (18.1%) and n = 447 (19.3%) among urban and rural residents, respectively.

By nationality, Kazakhs prevailed among the residents of the city in n = 2886 (65.6%) cases and Russians in n = 1028 (23.4%) cases. Also, among the respondents from the village, Kazakhs and Russians made up n = 1488 (64.2%) and n = 522 (22.5%), respectively.

According to marital status among urban and rural residents, married people prevailed, n = 2781 (63.2%) vs. n = 1666 (71.8%). While, single/single in n = 1148 (26.1%) cases were more common among study participants from the city, in contrast to n = 379 (16.3%) participants from the village.

In terms of smoking, in the majority of n = 941 (21.4%) cases, urban study participants were smokers compared to only n = 343 (14.8%) rural study participants who smoked.

According to the respondents' answers regarding the question about the frequency of drinking at least 1 dose of alcohol over the past 12 months, only 0.3% of respondents from urban and rural areas noted the daily use of alcohol, and 0.2% drank alcohol 5–6 days a week. The number of respondents drinking alcohol 1–2 times a week was almost twice as high among n = 974 (42.0%) urban residents compared to n = 974 (42.0%) rural survey participants. Also, respondents who drink alcohol 1–3 times a month were almost half as many among n = 151 (6.5%) rural residents compared to n = 521 (11.8%) urban survey participants. The number of respondents from the city equal to n = 1243 (28.2%) who drink alcohol less than once a month was relatively higher than the data of n = 509 (21.9%) in rural areas.

In terms of the presence of chronic kidney disease, according to the results of measuring eGFR, in most cases, participants in the study from the city and from the village had a normal eGFR equal to n = 3294 (74.8%) and n = 1647 (71.0%), respectively. While n = 636 (27.4%) residents of the village had a mild degree of CKD, which was higher than n = 1060 (24.1%) of this indicator among residents of the city. Also, in study participants from the village, CKD with eGFR < 60 ml/min/1.7 m^2^ was detected in n = 30 (1.3%) cases, which was higher than n = 40 (0.9%) cases identified in urban residents of this category CKD.

Thus, among all participants, regardless of place of residence, n = 4940 (73.5%) cases had normal eGFR, and n = 1695 (25.2%) participants had mild CKD (eGFR = 60–89 ml/min/1, 7 m^2^). CKD with eGFR < 60 ml/min/1.7 m^2^ was identified in n = 85 (1.3%) cases.

**Figure 1:**
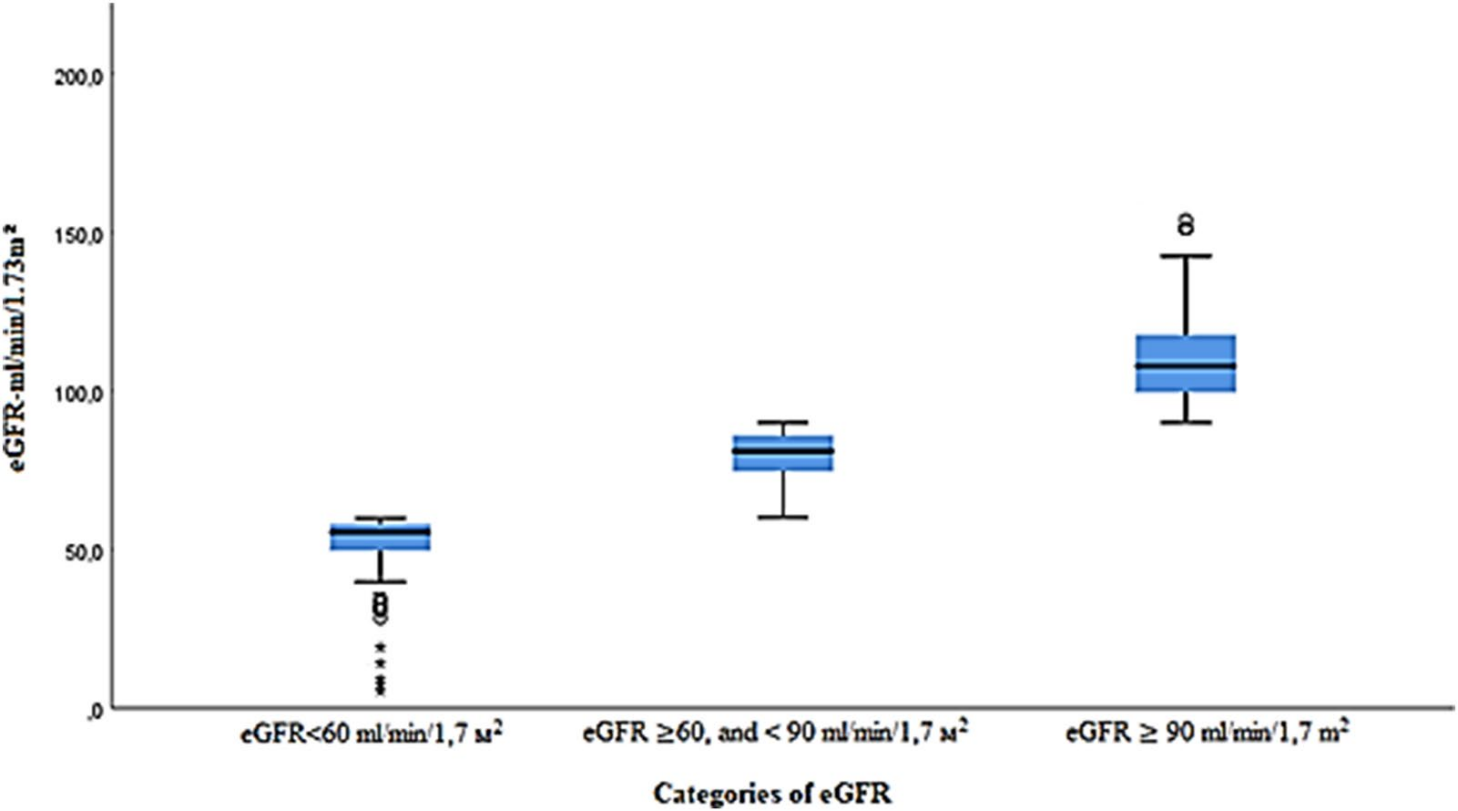
Characteristics of study participants by eGFR category.

In the total sample and according to the CKD-EPI formula, only 85 cases (1.3%; 95% CI 1.0–1.6%) had an eGFR < 60 ml/min/1.7 m^2^ consistent with the definition of CKD. However, 1695 cases (25.2%; 95% CI 24.2–26.3%) had eGFR ≥ 60 but < 90 mL/min/1.7 m^2^. The remaining 4940 cases (73.5%; 95% CI 72.4–74.6%) had ≥ 90 mL/min/1.7 m^2^ (Fig. 1).

It should be noted that the study identified 15 (0.2%) people with eGFR < 45 ml/min/1.7 m^2^. Of these, n = 9 (0.13%) were stage 3B (eGFR 30–44), n = 2 (0.02%) stage 4 (eGFR 15–29), and n = 4 (0.05%) stage 5 (eGFR below 15). All n = 15 respondents were phoned, of which all 15 confirmed that they were on dialysis.

**Figure 2:**
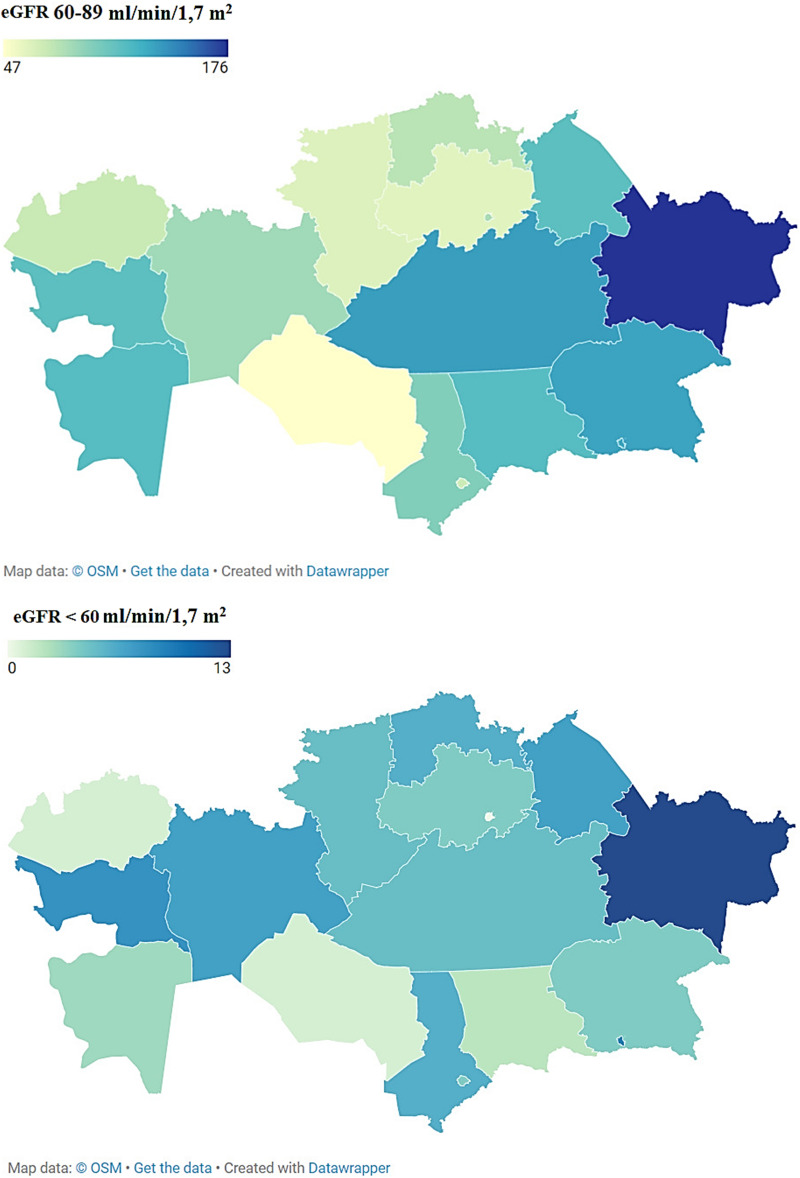
Prevalence of mild (eGFR = 60–89 ml/min) and CKD with eGFR < 60 ml/min/1.7 m^2^ over the study period throughout Kazakhstan (created via Datawrapper; available at https://www.datawrapper.de/maps).

The prevalence rates of mild (GFR = 60–89 ml/min/1.7 m^2^) and CKD with eGFR < 60 ml/min/1.7 m^2^ throughout Kazakhstan are shown in Fig. 2. According to the results, mild CKD (eGFR = 60–89 ml/min/1.7 m^2^) was most often determined in residents of the East Kazakhstan region in n = 176 (10.4%) cases, and also often in 7.8–8.0% of cases was registered in participants from Almaty and Karaganda regions. The least mild degree of CKD was characteristic of residents of Kyzylorda and Akmola regions with n = 47 (2.8%) and n = 61 (3.6%), respectively. CKD with eGFR < 60 ml/min/1.7 m^2^ in the majority of n = 13 (15.3%) and n = 9 (10.6%) cases was determined in study participants from the East Kazakhstan region and Almaty city, respectively. Also, in n = 8 (9.4%) cases, a moderate degree of CKD was registered among residents of the Atyrau region, while the smallest number of cases of a moderate degree of CKD equal to n = 1 (1.2%) were identified in the Western Kazakhstan and Kyzylorda regions.

Baseline characteristics of the study population based on eGFR results are presented in Table 2. According to the results, the mean age was the highest in subjects with eGFR < 60 ml/min/1.7 m^2^ at 56.8 ± 11.8 years compared with the mean age of participants with normal eGFR (36.7 ± 12.3 years), which was regarded as a statistically significant difference (p < 0.001). In addition, with eGFR < 60 ml/min/1.7 m^2^, the prevailing n = 67 (78.8%) study participants were in the older age category 50–69 years, which was also characteristic of n = 1043 (61.5%) study subjects with mild CKD. degree (p < 0.001). While almost half of study participants with normal eGFR were from the age group of 30–49 years, and these differences were statistically significant (p < 0.001).

**Table 2:** Baseline characteristics of the study population based on eGFR results.

Indicator n (%)	eGFR categories, ml/min/1.7 m^2^			p
	< 60 (n-85)	60–90 (n-1695)	90 and > (n-4940)	
Age average ± SD	56.8 ± 11.8	51.5 ± 11.7	36,7 ± 12.3	< 0.001*
18–29	4 (4.7)	87 (5.1)	1637 (33.1)	< 0.001*
30–49	14 (16.5)	565 (33.3)	2419 (49.0)	
50–69	67 (78.8)	1043 (61.5)	884 (17.9)	
Gender				
Male	30 (35.3)	731 (43.1)	2604 (52.7)	0.90
Female	55(64.7)	964 (56.9)	2336 (47.3)	
Site				
Urban	49 (57.6)	1058 (62.4)	3294 (66.7)	0.62
Rural	36 (42.4)	637 (37.6)	1646 (33.3)	
Education				
No school education	1 (1.2)	15 (0.9)	56 (1.1)	0.04*
Completed elementary (4 grd)	0 (0)	3 (0.2)	8 (0.2)	
Finished secondary (9 grd)	7 (8.2)	110 (6.5)	299 (6.1)	
Finished secondary (11 grd)	22 (25.9)	472 (27.8)	1297 (26.2)	
Higher	38 (44.7)	795 (46.9)	2239 (45.3)	
Master/Postgraduate/Doctorate	15 (17.6)	278 (16.4)	951 (19.2)	
7	1 (1.2)	9 (0.5)	66 (1.3)	
Refuses to answer	1 (1.2)	13 (0.8)	24 (0.5)	
Nationality				
Kazakh	38 (44.7)	874 (51.6)	3462 (70.1)	< 0.001*
Russian	34 (40.0)	594 (35.0)	922 (18.7)	
Uzbeks	2 (2.3)	36 (2.1)	164 (3.3)	
Ukrainians	0 (0)	50 (2.9)	60 (1.2)	
Uighurs	0 (0)	12 (0.7)	25 (0.5)	
Tatars	5 (5.9)	37 (2.2)	75 (1.5)	
Other	6 (7.1)	91 (5.4)	226 (4.6)	
Refuses to answer	0 (0)	1 (0.1)	6 (0.1)	
Family status				
Single, not married	6 (7.1)	190 (11.2)	1331 (26.9)	< 0.001*
Married	62 (72.9)	1199 (70.7)	3186 (64.5)	
Married/married but living separately	1 (1.2)	18 (1.1)	35 (0.7)	
Divorced	3 (3.5)	141 (8.3)	242 (4.9)	
Widower/widow	11 (12.9)	128 (7.5)	86 (1.7)	
Is in a civil marriage	2 (2.3)	19 (1.1)	51 (1.0)	
Refuses to answer	0 (0)	0 (0)	9 (0.2)	
Occupation				
State employee	8 (9.4)	192 (11.3)	653 (13.2)	< 0.001*
Private sector worker	18 (21.2)	554 (32.7)	2012 (40.7)	
Budget employee	12 (14.1)	200 (11.8)	608 (12.3)	
Entrepreneur	3 (3.5)	143 (8.4)	406 (8.2)	
Agricultural worker	1 (1.2)	10 (0.6)	39 (0.8)	
Student	0 (0)	9 (0.5)	282 (5.7)	
Housewife	2 (2.3)	109 (6.4)	390 (7.9)	
Pensioner	38 (44.7)	399 (23.5)	211 (4.3)	
Unemployed (able to work)	1 (1.2)	56 (3.3)	277 (5.6)	
Unemployed (unable to work)	2 (2.3)	20 (1.2)	36 (0.7)	
Refuses to answer	0 (0)	3 (0.2)	26 (0.5)	
BMI				
Underweight	3 (3.5)	117 (6.9)	459 (9.3)	0.02*
Normal weight	23 (27.0)	550 (32.4)	1587 (32.1)	
Overweight	30 (35.3)	28.7	1477 (29.9)	
Obesity I degree	16 (18.8)	307 (18.1)	893 (18.1)	
Obesity II degree	13 (15.3)	234 (13.8)	524 (10.6)	
Physical activity				
Yes	14 (16.5)	316 (18.6)	1082 (21.9)	0.04*
No	71 (83.5)	1379 (81.4)	3858 (78.1)	
Smoking				
Yes	2 (2.3)	254 (0)	1028 (20.8)	< 0.001*
No	83 (97.7)	1441 (0)	3912 (79.2)	
In the past 12 months, how often have you had at least 1 standard drink of alcohol?				
Daily	0 (0)	6 (14.9)	14 (0.3)	< 0.001*
5–6 days a week	0 (0)	4 (0.2)	9 (0.2)	
3–4 days a week	1 (1.2)	12 (0.7)	34 (0.7)	
1–2 days a week	2 (2.3)	92 (5.4)	264 (5.3)	
1–3 days per month	6 (7.1)	187 (11.0)	479 (9.7)	
Less than once a month/Holidays	19 (22.3)	479 (28.3)	1254 (25.4)	
Don't know	57 (67.0)	915 (53.9)	2886 (58.4)	
Do you currently smoke tobacco products daily?				
Yes	2 (2.3)	242 (14.3)	926 (18.7)	< 0.001*
No	0 (0)	35 (2.1)	177 (3.6)	
Don't know	83 (97.7)	1418 (83.6)	3837 (77.7)	

However, no statistically significant differences were found in eGFR depending on gender (p = 0.903) and place of residence (p = 0.62). Some statistically significant differences in education were observed between groups with different levels of eGFR (p = 0.04). The distribution of participants by nationality differed between groups with different levels of eGFR (p < 0.001). There were differences in the distribution of family status between groups with different levels of eGFR (p < 0.001). The distribution of employment differed between groups with different levels of eGFR (p < 0.001). Differences were found in the distribution of BMI between groups with different levels of eGFR (p < 0.02). Differences in physical activity were noticeable between groups with different levels of eGFR (p = 0.04). The distribution of smoking differed between groups with different levels of eGFR (p < 0.001). Significant differences were observed in the frequency of alcohol consumption between groups with different levels of eGFR (p < 0.001).

The results of the analysis of the use of logistic regression models to determine the co-factors of kidney dysfunction are presented in Table 3. In the rough model, there is a statistically significant association (p = 0.001) between urban or rural residence and kidney dysfunction. However, after adjusting the model, this relationship becomes insignificant (p = 0.23).

**Table 3:** Logistic regression (rough and adjusted) of factors influencing the risk of developing CKD in the study subjects.

Indicator	Corrected model 1				Corrected model 2			
	OR	95% confidence interval for		Value	OR	95% confidence interval for		Value
		Low	High			Low	High	
City/Village	0.75	0.63	0.89	0.001	1.09	0.94	1.26	0.23
Astana city	1.14	0.75	1.73	0.53	0.86	0.59	1.24	0.42
Almaty city	0.97	0.65	1.44	0.88	0.60	0.43	0.85	0.001
Akmola region	1.83	1.16	2.89	0.001	1.00	0.67	1.50	0.99
Aktobe region	0.61	0.39	0.93	0.02	0.49	0.34	0.72	0.001
Alma-Ata's region	0.76	0.51	1.13	0.17	0.74	0.52	1.06	0.09
Atyrau region	0.25	0.17	0.37	0.001	0.31	0.22	0.45	0.001
West-Kazakhstan region	0.50	0.32	0.79	0.001	0.43	0.29	0.65	0.001
Jambyl Region	0.65	0.43	0.98	0.04	0.59	0.42	0.86	0.001
Karaganda region	0.80	0.53	1.21	0.30	0.47	0.33	0.66	0.001
Kostanay region	1.59	1.03	2.49	0.04	1.00	0.67	1.49	0.99
Kyzylorda Region	1.22	0.77	1.93	0.39	1.28	0.83	1.95	0.26
Mangistau region	0.33	0.22	0.50	0.001	0.33	0.23	0.48	1.56
Turkestan region	0.75	0.50	1.13	0.17	0.89	0.62	1.29	0.55
Pavlodar region	0.56	0.37	0.86	0.001	0.34	0.23	0.49	1.05
North-Kazakhstan region	0.60	0.37	0.96	0.03	0.39	0.26	0.59	2.54
East Kazakhstan region	0.33	0.23	0.49	0.001	0.27	0.19	0.38	1.36
Shymkent city	0.29	0.06	1.32	0.11	0.29	0.09	0.99	0.05
Smoking	1.43	0.73	2.79	0.29	1.21	0.72	2.04	0.46
Drinking alcohol 3–4 days per week in the last 12 months	0.71	0.24	2.05	0.04	0.85	0.62	1.16	0.31
Physical activity	0.31	0.07	1.41	0.13	1.08	0.93	1.25	0.34
Underweight	0.35	0.07	1.89	0.02	1.43	1.09	1.88	0.01
Normal weight	0.37	0.07	1.92	0.02	1.10	0.91	1.34	0.32
Overweight	0.32	0.07	1.45	0.01	1.18	0.97	1.44	0.10
Obesity	0.31	0.07	1.39	0.27	1.24	0.99	1.53	0.04

Significant values are in bold.

By region, in a rough model, some regions have a statistically significant association with impaired renal function. After adjusting the model, some of these associations remain statistically significant, while others become insignificant.

In a rough model, the relationship with residence in the territory of Akmola region (OR 1.83, 95% CI (1.16–2.89), p = 0.001), Aktobe region (OR 0.606, 95% CI (0.395–0.928), p = 0.021), Atyrau region (OR 0.25, 95% CI (0.167–0.37), p = 0.001), West Kazakhstan region (OR 0.50, 95% CI (0.32–0.79), p = 0.001), Zhambyl region (OR 0.65, 95% CI (0.43–0.98), p = 0.04), Kostanay region (OR 1.59, 95% CI (1.03–2.49), p = 0.04), Mangystau region (OR 0.33, 95% CI (0.22–0.50), p = 0.001), Pavlodar region (OR 0.56, 95% CI (0.37–0.86), p = 0.001), North Kazakhstan region (OR 0.60, 95% CI (0.37–0.96), p = 0.03), and East Kazakhstan region (OR 0.33, 95% CI (0.23–0.49), p = 0.001).

In the adjusted model, a statistically significant relationship was found between the development of CKD and residence in Almaty city (OR 0.60, 95% CI (0.43–0.85), p = 0.001), Aktobe region (OR 0.49, 95% CI (0.34–0.72), p = 0.001), Atyrau region (OR 0.31, 95% CI (0.22–0.45), p = 0.001), West Kazakhstan region (OR 0.43, 95% CI (0.29–0.65), p = 0.001), Karagandy region (OR 0.47, 95% CI (0.33–0.66), p = 0.001), Zhambyl region (OR 0.59, 95% CI (0.42–0.86), p = 0.001), and Shymkent city (OR 0.29, 95% CI (0.09–0.99), p = 0.05).

For the smoking factor, in a rough model, the association between smoking and impaired renal function is not statistically significant (p = 0.29). After adjusting the model, the relationship remains insignificant (p = 0.46).

For alcohol consumption with a frequency of 3–4 days per week, in a rough model, the relationship is statistically significant (p = 0.04). However, after adjusting the model, the relationship becomes insignificant (p = 0.31).

For physical activity, in a crude model, the association between physical activity and impaired renal function is not statistically significant (p = 0.13). After adjusting the model, the relationship remains insignificant (p = 0.34).

Based on the presence of underweight, in a rough model, the association between underweight and impaired renal function is statistically significant (p = 0.02). After adjusting the model, the relationship also remains significant (p = 0.01).

In the presence of normal weight, in a rough model, the association between normal weight and impaired renal function is statistically significant (p = 0.02). However, after adjusting the model, the relationship becomes insignificant (p = 0.32).

In terms of being overweight, in a rough model the association between overweight and impaired renal function is statistically significant (p = 0.01). However, after adjusting the model, the relationship becomes insignificant (p = 0.10).

Consideration of obesity as a potential risk factor showed that, in a rough model, the association between obesity and impaired renal function is not statistically significant (p = 0.27). However, after adjusting the model, the relationship becomes significant (p = 0.04).

These results underscore the importance of considering confounding factors when analyzing the association between various factors and impaired renal function.

## Discussion

The aim of this study was to determine the population prevalence of CKD and related factors in Kazakhstan. Despite the existence of localized studies conducted in Kazakhstan with Kazakh patients with CKD^13^, the true prevalence of this disease has not been determined. However, it is worth noting that in a study of the prevalence, morbidity and mortality of patients on dialysis in Kazakhstan^14^. There is consistency in the data with some of our indicators, for example, the prevalence of patients older than 50 years of age, as well as the similarity of data on the national composition.

As is well known, the results of a nationwide survey with a unique set of data that can examine the impact of many socio-demographic variables on the burden and trends of CKD could be demonstrative data for determining the prevalence of this disease. Due to the asymptomatic nature of the disease, CKD is often not detected until late in its progression, resulting in lost opportunities for prevention. The progression of kidney failure or other adverse outcomes can be prevented or delayed by early detection and treatment of CKD^15^.

Decrease in eGFR < 60 ml/min/1.7 m^2^ is considered as a sign of CKD, as well as structural or functional impairment of kidney function^16^.

In our study, the prevalence of CKD with eGFR < 60 ml/min/1.7 m^2^ was determined in 1.3% of cases. This prevalence rate, compared with global prevalence rates, is considered to be rather low. For example, the global prevalence of CKD is 11–13%^17^. In Asia, home to 60% of the world's population, the prevalence of CKD is reported to be one of the highest in the world^18^. CKD prevalence of 11% has been reported in several regions of China^19^.

However, in this study, 25.2% of participants had mild CKD. And in the vast majority of cases, 73.5% of cases had normal eGFR.

In study participants from the village, CKD with eGFR < 60 ml/min/1.7 m^2^ was detected in 1.3% of cases, which was higher than 0.9% of cases of moderate CKD detected in urban residents. Data published in other studies suggest that exposure to some of the putative potential risk factors for CKD, such as agricultural work and exposure to agrochemicals, among others, may increase the incidence of CKD and be more significant in rural areas^20^.

According to the results, a mild degree of CKD was most often detected in residents of the East Kazakhstan region in 10.4% of cases, and also often in 7.8–8.0% of cases it was registered in participants from Almaty and Karaganda regions. CKD with eGFR < 60 ml/min/1.7 m^2^ in the majority of 15.3% of cases was registered in residents of the East Kazakhstan region, 10.6% of cases were detected in study participants from and the city of Almaty, and in 9.4% of cases it was registered in residents of Atyrau region.

The relatively high rates of CKD registration among study participants living in the East Kazakhstan region may be due to the environmental, namely the state of the air and water basins, and the agricultural activities of this region^21^. Wasteful attitude to the use of land, pollution with pesticides, waterlogging of the soil, violations that contribute to the development of water and wind erosion, lead to a decrease in fertility and a reduction in usable areas. The use of highly toxic pesticides in almost all farms in the region contributes to the accumulation of persistent pesticides in soils. In addition, the characteristic of water quality in the rivers of the East Kazakhstan region corresponds to “high” and “extremely high” levels of pollution^21^. According to literature sources, in the world practice, the global epidemic of CKD of unknown etiology was determined mainly in the agricultural communities of some countries^22^. And this fact may have been associated with the widespread use of fertilizers, which are used in the field to increase crop yields, also containing phosphates and nitrates^23^. Despite this, the role of nephrotoxic agrochemicals in the aetiology of CKD and the extent of their contribution to the CKD epidemic, if any, cannot be adequately assessed based on the currently available data requiring further research^24^.

Age is one of the most important factors influencing kidney function, and kidney function is generally stable from infancy to late adulthood^25^. However, it is known that CKD affects all age groups and in most cases does not depend on sex, and is more common in the elderly. Previously estimated global prevalence of this disease is 23–36% in people aged ≥ 64 years^1^. According to our results, in CKD with eGFR < 60 ml/min/1.7 m^2^, the prevailing proportion of 78.8% of the study participants were in the older age category 50–69 years. Also, the age of those surveyed with mild CKD in 61.5% of cases was 50–69 years (p < 0.001). The decline in kidney function may be related to changes in the structure of the kidneys associated with aging, since in healthy people after 30 years of age, GFR decreases by 1 ml/min/1.7 m^2^ per year^26^.

Our study did not reveal statistically significant differences in eGFR depending on gender (p = 0.90) and place of residence (p = 0.62). However, according to global burden studies, the prevalence of CKD in women tends to be higher than in men in various countries^27^.

According to the results of our study, depending on the indicators of the level of eGFR, statistically significant differences were also found depending on the level of education, nationality, marital status, employment, BMI, physical activity, the fact of smoking, and alcohol consumption (p ≤ 0.05). Given the multinational composition of Kazakhstan, the possibility of the influence of ethnicity as a factor associated with CKD was also studied, however, no statistically significant differences were found during the regression analysis, which requires further research in this area. Since the mechanism underlying these differences may be multifactorial, including cultural differences such as smoking habits, alcohol consumption, lifestyle and genetic factors^28^.

Previously published data suggest that hypertension, diabetes and obesity are among the growing noncommunicable diseases and are important risk factors for CKD^29^. In our study, when analysing the relationship between potential factors and the development of CKD, in addition to a certain relationship with the region of residence, with adjusted logistic regression, a statistically significant relationship was found between the risk of developing CKD and underweight, as well as the presence of obesity (p ≤ 0.05). A growing body of research indicates that obesity is a driver of CKD, and the mechanisms behind this are complex and include hemodynamic changes, inflammation, oxidative stress, and activation of the renin–angiotensin–aldosterone system^30^.

Importantly, screening for kidney disease and awareness of the risks associated with it are key to the early detection and management of chronic kidney disease, and it is also essential to include them in the database for the development of a national prevention policy.

This fact shows the importance of the functioning of the national register of patients with CKD, since according to previously published data, low awareness of CKD and suboptimal screening for CKD may have contributed to the lack of data on the consequences of undiagnosed and untreated CKD for specific neighbouring countries, as well as for Kazakhstan^31^. In this regard, in the future there is a high need for the formation of early detection programs that can be used in the development of toolkits for CKD screening^32^.

Thus, the data obtained in our cross-sectional study on the prevalence of CKD, the severity and risk factors of this disease among the population of Kazakhstan, depending on the region of residence, can serve as a tool for the proper distribution of the workload on doctors in the future, as well as for the timely differentiation of a group of patients with CKD with indications for replacement therapy.

### Study strengths and limitations

The main advantage of the study was the use of random selection of participants based on the population and a large sample size (participants from all 14 regions and 3 major cities in Kazakhstan). In addition, a comprehensive assessment of the various sociodemographic factors associated with CKD may be of potential benefit, as this provides a more complete understanding of the determinants of the disease in a population. Moreover, including participants from both rural and urban areas allows comparison of CKD prevalence and associated risk factors between these settings. Nonetheless, this study has some limitations. The diagnosis of CKD was based on a single eGFR assessment, without albumin testing, which may tend to overestimate the incidence of kidney disease. Moreover, estimated GFRs show a high degree of inter-individual variability and ideally require repeat measurements to accurately represent kidney function at least 3 months later.

The results indicate that during the study period, the prevalence of CKD with eGFR < 60 ml/min/1.7 m^2^ was at the level of 1.3%. Apart from that, it is also worth noting the fact that a fairly large part of the study subjects had mild CKD (25.2%). In addition, statistically significant correlations of the risk of developing CKD with such factors as the region of residence, underweight, and obesity were determined.

The obtained data on the prevalence of CKD can serve as a tool for the proper distribution of the workload on doctors and the timely provision of care to patients with CKD. However, due to the relatively low number of cases of CKD with eGFR < 60 ml/min/1.7 m^2^, this cross-sectional study cannot reflect the trend in the prevalence of this disease in Kazakhstan. Therefore, further intensive research is necessary to investigate the changes in the prevalence of CKD over a longer period.

## Methods

### Ethical issues

The study was approved by the Local Ethics Committee of the S.D. Asfendiyarov Kazakh National Medical University, Almaty, Republic of Kazakhstan (protocol of the Local Ethics Commission No. 12 (118) dated 28.09.2021). In addition, this investigation also was approved by the Central Bioethics Commission of Ministry of Healthcare of the Republic of Kazakhstan (protocol No. 14 dated 24.11.2021). Moreover, the study was registered with ClinicalTrials.gov (NCT05122832). All methods were performed in accordance with the relevant guidelines. Informed consent was obtained from all subjects and/or their legal guardians.

### Study setting

Kazakhstan is located in the Central Asia. It is administratively divided into 14 regions with 177 districts and cities. In addition, Kazakhstan has three cities of “republican significance”: Astana, (the capital city, previously known as ‘Nur-Sultan’), Almaty, the former capital city, and Shymkent, the third largest city in KZ. In general, urban areas are considered as the town or city and rural areas as the district. The country’s population is around 20 million people, and the population density is 6 people per square kilometer. The majority of inhabitants reside in urban areas.

### Inclusion criteria

For this study based on WHO STEPS questionnaire inclusion criteria were as follows: participants aged 18 and 69 years, both male and female, who were residents of the surveyed regions in Kazakhstan.

### Exclusion criteria

For this study were as follows: involved individuals who were unable to provide informed consent due to cognitive impairment or any other reason that could compromise their ability to understand the study procedures. Additionally, individuals who were not residents of the surveyed regions, or those unwilling to participate in the study were also excluded.

### Study design and population

This cross-sectional study consisted of a representative sample of people aged 18 years and over in the general population of the population of Kazakhstan for the period October 2021 to May 2022 from 14 regions, also additionally including large metropolitan cities such as Almaty, Astana and Shymkent.

6,720 people aged 18 to 69 were recruited throughout Kazakhstan. Participation in the study was completely voluntary.

### Sampling

We used weighted, multistage, cluster sampling method and included 8 groups with a division into 4 age groups—18–29 years, 30–44 years, 45–59 years, 60–69 years, as well as with stratification by sex (men and women) in each age group. Study sample size determined using WHO's special STEPS tool (sample_size_calculator Excel format) using the following methodology:

Probability value for 95% confidence interval—1.96;

Estimated prevalence of the risk factors—0.5;

Margin of error—0.05;

Design effect—1.5;

Anticipated response rate—70%

The preliminary calculation resulted in the sample size of n = 6585.

The multistage cluster sampling in this study has three levels, with clustering occurring at each level. At the first stage, we selected the primary sampling units: districts and cities. The primary sampling units (clusters) were proportionally selected among all economic regions. Information about districts and cities (Almaty, Astana and Shymkent cities) and all 14 regions was received from the Bureau of National statistics, Agency for Strategic planning and reforms of the Republic of Kazakhstan (https://stat.gov.kz/).

At the second stage, we selected the secondary sampling units (SSU) of Primary Health Care facilities (PHC), which provide medical care for local population. For a selection of SSUs, we used data from the Republican state enterprise on the right of economic management "Republican Centre for Healthcare Development" of the Ministry of Health of the Republic of Kazakhstan (RCHD) (https://stat.gov.kz/). A register of PHC facilities was obtained with an indication of the number of people served. SSUs were selected by random sampling method and with a probability proportional to the number of populations served in each PHC facility.

At the third stage, we selected the tertiary sampling units: households and respondents. The size of households per PHC facility was calculated using the following formula:

Household size per PHC facility = 6585 / 240 ≈ 28.

Then we calculated final total sample size: Final Total Sample Size = 240 × 28 = 6720.

For the selection of households, a list of households served by chosen PHC facilities was obtained.

Households were randomly selected from each facility using the Randhold.xls tool to participate in the study. The final selection of respondents aged 18–69 from each selected household was carried out using the Kish method. This selection method was carried out according to a special methodology, including random selection of the respondent depending on the sex and age of all residents of the household that meet the criteria for inclusion in this study.

The participation rate in the study was 95%. This high level of participation may be attributed to the participants being fully informed about the study's objectives and understanding its significance. Furthermore, the use of local laboratories (which the participants trust) may have contributed to the high participation rate. Additionally, it's worth noting that the research was conducted at times and locations convenient for the study participants.

### Survey

The Russian translated version of the STEPS questionnaire, which was translated previously (WHO STEPS tool (basic and advanced modules) were used^33^. The WHO STEPS questionnaires were uploaded to the HealthTrack mobile app^34^ for further use by the interviewers. All interviewers who conducted the survey were certified.

### Covariates

Blood pressure was measured by means of three tests, unless the difference between the readings exceeded 10 mm Hg. Art. In this case the average value of the two closest measurements was used. The measurement was performed using an Omron digital automatic blood pressure monitor model HEM-8712 (Omron Health Care Co., Japan) with cuffs of the appropriate size^28^.

Elevated blood pressure was defined as systolic blood pressure ≥ 140 mm Hg. Art. and/or diastolic blood pressure ≥ 90 mm Hg. Art. during the study or as previously diagnosed arterial hypertension.

BMI indicators were divided into 5 categories: BMI < 18.4—underweight, BMI >  = 18.5 and < 24.9—normal weight, BMI >  = 25 and < 29.9—overweight, BMI >  = 30 and < 34, 9—obesity of the 1st degree, BMI > 35—obesity of the 2nd degree.

According to the level of education, the respondents were divided into the following groups: no school education; completed primary (grade 4); completed secondary (grade 9); completed secondary (grade 11); higher; master's/postgraduate/doctoral studies; refuses to answer.

By nationality, the respondents were divided into the following groups: Kazakhs, Russians, Uzbeks, Ukrainians, Uighurs, Tatars, others, and those who refused to answer.

According to marital status, there was a division into the following categories: single/not married; married; married/married, but lives separately; divorced; widower/widow; is in a civil marriage; and those who refused to answer.

According to the smoking factor, the respondents were divided into smokers and non-smokers.

Regarding alcohol consumption, when asked about the frequency of drinking at least 1 dose of alcohol over the past 12 months, the respondents' answers were divided into several categories: daily; 5–6 days a week; 3–4 days a week; 1–2 days a week; 1–3 days per month; less than once a month/holidays; and those who refused to answer.

To measure eGFR, blood was taken by veno-puncture after an overnight fast of at least 10 h. Serum creatinine was measured by the same methods. We used the CKD-EPI equations to determine the glomerular filtration rate (GFR)^35^.

The CKD-EPI (creatinine) score was developed in 2009^36^, when it was shown that the CKD-EPI creatinine equation is more accurate than the Modification of Diet in Renal Disease Study equation and can replace it for routine clinical use.

Depending on the glomerular filtration rate, the degree of CKD was divided into 5 stages: stage 1—with normal or high eGFR (eGFR > 90 ml/min/1.7 m^2^); stage 2—mild CKD (eGFR = 60–89 ml/min/1.7 m^2^); stage 3A—moderate CKD (eGFR = 45–59 ml/min/1.7 m^2^); stage 3B—moderate CKD (eGFR = 30–44 ml/min/1.7 m^2^); stage 4—severe CKD (eGFR = 15–29 ml/min/1.7 m^2^); stage 5—end-stage CKD (eGFR < 15 ml/min/1.7 m^2^).

### Statistical analysis

Statistical analysis was conducted using SPSS software (version 25.0, IBM SPSS Inc., Chicago, Illinois, USA). Categorical variables were expressed in terms of frequencies (n) and percentages (%). Quantitative variables were expressed in terms of mean and standard deviation (SD). The distributions of variables were evaluated using a histogram, a quantile–quantile plot, and the Kolmogorov–Smirnov test. Since very few cases were found with GFR levels consistent with CKD (i.e. eGFR < 60 mL/min/1.7 m^2^), we analysed factors associated with impaired renal function (eGFR < 90 mL/min/1, 7 m^2^).

We used logistic regression models to determine concomitant factors for impaired renal function. As a result, a rough model and adjusted models are shown. The first adjusted regression model controlled for age (as a continuous variable) and gender; while the second regression model also included ethical affiliation, status, and educational attainment as potential confounders.

## Data Availability

All datasets used and analysed in this study are available from the corresponding author on request.
